# Socio-demographic Risk Factors of Gestational Diabetes Mellitus

**DOI:** 10.12669/pjms.293.3629

**Published:** 2013

**Authors:** Radhia Khan, Khurshid Ali, Zakkia Khan

**Affiliations:** 1 Radhia Khan, M.Phil Biochemistry, Department of Biochemistry, Khyber Girls Medical College, Peshawar, Pakistan.; 2 Prof. Dr. Khurshid Ali, PhD Chemistry, Institute of Chemical Sciences, University of Peshawar, Pakistan.; 3 Dr. Zakkia Khan, MBBS, FCPS, Medical Officer, Gynea Ward, Khyber Teaching Hospital, Peshawar, Pakistan.

**Keywords:** Gestational diabetes, parity, BMI and socioecnomic status

## Abstract

***Objective: ***The objective of the study was to report the socio demographic risk factors of gestational diabetes mellitus (GDM).

***Methods: ***This study was conducted in the Institute of Chemical Sciences, University of Peshawar. In this study 103 GDM and 97 healthy pregnant women (HPW) were registered in Khyber Teaching Hospital (KTH), Peshawar, Pakistan. Women with gestational diabetes were diagnosed with 75mg Oral Glucose Tolerance Test (OGTT). Data was collected through questionnaire which had information about sociodemographic risk factors.

***Results: ***Maternal age, BMI and parity of GDM were significantly higher at P<0.05 as compared to HPW. Previous history of gestational diabetes and family history of diabetes of GDM women were also significantly higher at P<0.001 as compared the control group. Socioecnomic status, education level and occupations of GDM and HPW were not significantly different.

***Conclusion: ***Maternal age, BMI, parity, previous history of gestational diabetes and family history of diabetes are the high risk factors of GDM. Socioecnomic status does not affect the prevalence of GDM.

## INTRODUCTION

Pregnancy is a unique normal physiological state where life exists on life. The fetus is entirely dependent on mother for its healthy growth. To keep the fetus growing and the mother healthy, the body of the pregnant woman has to make biochemical and physiological changes in cardiovascular, hematological, renal, metabolic and respiratory systems. All these changes are required in normal pregnancy and during the complications of pregnancy. To cope with the new environment of pregnancy, the body increases its blood sugar, cardiac output and breathing rate. Levels of progesterone, estrogen, cortisol and prolactin also rise continuously throughout normal pregnancy to provide better and nourished environment to the fetus. The biochemical changes during pregnancy result in accumulation of lipid in early gestation, which results in insulin resistance and metabolic syndrome. Metabolic syndrome is a condition which involves central obesity and any two of the following factors; raised triglyceride levels, reduced high density lipoprotein (HDL) cholesterol, raised blood pressure and raised fasting plasma glucose. In the altered physiological state of pregnant woman, many complications can occur. Amongst these complications gestational diabetes, preclampsia, hypertension and obesity are the most serious and fatal ones associated with pregnancy. Gestational diabetes mellitus (GDM) is a condition of pregnant woman where glucose intolerance is found during pregnancy. GDM usually develops during pregnancy and ends after pregnancy.^1^

In US the incidence of GDM is reported 14% of all pregnancies and the rate of incidence is constantly increasing in multiethnic populations.^2^ GDM is one of the well-known risk factor for developing type 2 diabetes in future.^3^ The various factors that will predict the pregnant woman to become diabetic in future are: early diagnosis of GDM in pregnancy, need for insulin treatment during pregnancy, high blood glucose levels at diagnosis, preterm delivery, macrosomic babies and an abnormal oral glucose tolerance test after two months of delivery.^4^ Recently, it has been reported that GDM has strong association with increased risk of serious perinatal morbidities and mortalities, as well as maternal morbidities.^5^ Gestational diabetic women are at high risk of pre-eclampsia, hypertension, preterm deliveries, caesarian section, still births and insulin treatment. Neonates of the gestational diabetic mothers are usually big in size and large for gestational age.^6^ GDM mothers should be examined and diagnosed during early pregnancy and they should have regular postpartum check up for recognition and management of complications.

The prevalence of GDM has weak but significant relation with socioeconomic status including education level, ethnicity, parity, maternal age, smoking, nutrition, previous history of GDM and family history of diabetes.^7^ The association between GDM and socioeconomic status is not well established because previous studies have reported conflicting results due to different definitions used for economic status. The increasing incidence of GDM, independent of ethnicity, socioeconomic status, or maternal age, has many short-term adverse pregnancy outcomes and long-term future risk of type 2 diabetes. Smoking has been suggested as the key factor underlying socioeconomic differences in low birth weight and infant mortality. Lower socioeconomic status is well recognized as a risk for chronic disease in developed and developing countries. Socially disadvantaged GDM women are less likely to seek perinatal care and thus having more pregnancy complications.^8^

The factors already reported to influence the risk of GDM among mothers are previous history of GDM, family history of diabetes, obesity, recurrent urinary tract infections, infertility treatment, unexplained neonatal death, macrosomic babies, prematurity, pre-eclampsia and advanced maternal age.^9^^-^^10^ GDM is a disorder which can be effectively controlled by decreasing the high risk factors and thus leading to healthy infant delivery. Thus accurate monitoring and proper management of GDM women will result in improved maternal and neonatal consequences.^11^^,^^12^ This study was conducted to report the socio demographic risk factors involved in the development of GDM and to educate high risk pregnant women to take proper measures to decrease morbidity and mortality of GDM.

## METHODS

This study was conducted in the Institute of Chemical Sciences, University of Peshawar from January 2012 to September 2012. In this comparative analytical study GDM and HPW for comparison were registered in Khyber Teaching Hospital (KTH), Peshawar, Pakistan. Information was collected from the registered women on well designed questionnaire. Those GDM and HPW were selected who were at the gestational age of ≥28 weeks and were not having previous history of medical illness like hypertension, cardiac and renal diseases. It was ensured that the selected pregnant women were not having any medical treatment that affects lipid profile and hormones concentration.

Sociodemographic data of the pregnant women were obtained during the face to face interview. During the study women were screened for GDM by determining both fasting and random blood glucose level. If the fasting blood glucose level was ≥ 105mg/dL and random blood glucose level was ≥ 140mg/dL, the pregnant women were identified for GDM. The identified GDM women then underwent 75g two hour oral glucose tolerance test for the confirmation of GDM. One hundred and ten GDM and one HPW were registered for the study. The HPW were used as a control group. Both the GDM and HPW were at gestational age of 28 weeks or more. The GDM were the admitted patients of Gynea Ward of Khyber Teaching Hospital, Peshawar, Pakistan. They were admitted for control of gestational diabetes or treatment of its complications. HPW who were not having any medical problem and were at the gestational age of 28 weeks or more were also registered. Consents from the registered pregnant women were obtained. Seven GDM patients and 3 HPW dropped from the study and the remaining 103 GDM patients and 97 HPW completed the study.

BMI was calculated from the height and weight of the registered pregnant women using the formula: BMI = Weight in Kg/ Height in (meters)^2^

Height of each registered pregnant woman was measured in standing position without shoes using vertical calibrated scale. Heel to head-crown length was measured in centimeters. Weight of each registered pregnant woman was taken in standing position without shoes using an accurate health weighing scale.

Well designed questionnaires were used to collect data from the registered pregnant women. Face to face interviews were conducted in the local language. The well designed questionnaires covered sociodemographic characteristics of the pregnant women, family and medical history, maternal and neonatal problems and complications. Statistical analysis were done by using SPSS computer software version 10. Chi-square test was performed to test for differences in the proportions of categorical variables between two or more groups. Student *t*-test (two tailed) was used to determine the significance. The level P < 0.05 was taken as the cut off value for significance.

## RESULTS

Table-I shows the sociodemographic risk factors in pregnant women with GDM and without GDM (HPW).The data indicated that out of 103 admitted GDM women 73.8% were Pakistani and 26.2% were non Pakistani. The data also showed that monthly income, female occupation and education level of GDM women were not significantly different from HPW. However, parity, family history of diabetes and previous history of GDM were significantly different among the two groups. The number of GDM women in grand multiparous group was 54.5% while HPW was 37.2%, P=0.05. Family history of diabetes was reported by 84.5% GDM and 26.8% HPW, P<0.001. Previous history of gestational diabetes was reported by 75.5% GDM. None of the HPW was diabetic in their previous pregnancies.

Data in Table-II shows that mean maternal age, mean BMI and mean parity of GDM women was significantly higher than the control. The mean maternal age of GDM and HPW was 35.01 ± 4.54 vs 31.29 ± 5.79 years, P<0.001, mean BMI was 28.03 ± 2.89 vs 27.29 ± 1.89 kg/m^2^ and mean parity was 5.63 ± 2.01 vs 4.95 ± 2.43, P=0.05.

## DISCUSSION

The dramatic increase in the prevalence of GDM and its adverse maternal and neonatal complications may possibly be reduced by controlling the risk factors involved in the development of GDM. According to the present study the high risk factors of GDM were advanced maternal age, increased BMI, parity, family history of diabetes and previous history of gestational diabetes. Our findings were in accordance with the findings of Ben-Haroush et al who had reported that maternal age, parity, smoking, obesity and family history of diabetes are the high risk factors for gestational diabetes.^13^

**Table-I:** Sociodemographic risk factors for GDM in Pakistan

*Variables*	*GDM (n=103)*	*HPW (n=97)*	*P-value*
*Nationality*			
Pakistani	76 (73.8%)	67 (69.1%)	NS (0.531)
Non Pakistani	27 (26.2%)	31 (30.9%)	
*Monthly Income groups*			
≤ Rs.15,000	15 (14.5%)	21 (21.6%)	NS (0.184)
Rs.15,001-30,000	44 (42.7%)	48 (49.4%)	
Rs.30,001-45,000	28 (27.2%)	16 (16.5%)	
> Rs.45,000	16 (15.5%)	12 (12.4%)	
*Occupation*			
House wife	78 (75.7%)	80 (82.4%)	NS (0.298)
Professional	25 (24.3%)	17 (17.5%)	
*Education Levels*			
Illiterate	62 (60.2%)	67 (69.1%)	NS (0.422)
School’s Education	25 (24.3%)	18 (18.5%)	
Above School’s Education	16 (15.5%)	12 (12.4%)	
*Parity *			
Primiparous	10 (9.7%)	13 (13.4%)	0.05
Multiparous	37 (35.9%)	48 (49.4%)	
Grand Multiparous	56 (54.4%)	36 (37.2%)	
*Family history of diabetes*			
Yes	87 (84.5%)	26 (26.8%)	< 0.001
No	16 (15.5%)	71 (73.2%)	
*Previous history of GDM*			
Yes	78 (75.5%)	00 (00%)	< 0.001
No	25 (24.3%)	97 (100%)

*GDM stands for gestational diabetes and HPW for healthy pregnant women*

*P-value determined by chi-square test is given in column 4*

**Table-II:** Mean ± SD values of sociodemograhic correlates in GDM and HPW

*Variables*	*GDM (n= 103)* *Mean ± SD*	*HPW (n=97)* *Mean ± SD*	*P-value*
Monthly income (Rs)	30845 ± 11107	28360 ±11511	NS (0.12)
Maternal age (years)	35.01 ± 4.54	31.29 ± 5.79	<0.001
BMI (kg/m^2^)	28.03 ± 2.89	27.29 ± 1.89	0.001
Parity	5.63 ± 2.01	4.95 ± 2.43	0.03

*GDM stands for Gestational diabetes mellitus and HPW stands for healthy pregnant women *

*Mean ± SD values of Monthly income, Maternal age, BMI and Parity of GDM and HPW*

The current study identified no significant association between socioeconomic status and GDM. Lower socioeconomic status is well recognized as a risk for chronic disease in developed and developing countries.^14^ The association between GDM and socioeconomic status is less well established, with conflicting results seen in previous studies. These studies cannot easily be compared because of different definitions of social status used, depending upon monthly income, educational attainment, employment, family influence, type of health care and house hold characteristics. Tanaka et al found no association, while Clausen et al showed that living in an area of deprivation was positively associated with GDM.^15^^,^^16^ Lower socioeconomic status is associated with an increased risk of various adverse pregnancy outcomes such as perinatal mortality, miscarriages, preterm birth, and lower birth weight. The education status of GDM and HPW were not significantly different. The study shows that 60.2% GDM women were illiterate and ignorant of the disease. The data in Table-I revealed that grand multiparous women were more prone to gestational diabetes as compared to HPW. The number of GDM women with grand multiparity was 54.4%, which was significantly higher at P=0.001 than the HPW. Family history of diabetes and previous history of gestational diabetes also plays an important role in the increase prevalence of GDM. GDM was more prevalent in women having family history of diabetes (84.5%) and in women who have been exposed to gestational diabetes (77.5%) in their previous pregnancies. Epidemiological studies have always identified increased multiparity, family history of diabetes in first degree relatives and previous history of gestational diabetes as high risk factors for the development of GDM.^17^^,^^18^

In this study a significant increase in the maternal age, BMI and parity of GDM women were observed. These results are similar to the previous study of Doherty et al.^19^ Thus obese pregnant women with increased maternal age should be conscious of all the risks of maternal obesity and advanced maternal age and of how it can affect their pregnancies. The major limitation of the study was that it was a hospital based study in which only the admitted GDM women were registered. It would be much better if the sample size is increased and follow up of GDM women until delivery are made to report the relationship between sociodemographic risk factors and adverse maternal and neonatal complications of gestational diabetes.

## CONCLUSION

Advanced maternal age, increased BMI, multi parity, previous history of gestational diabetes and family history of diabetes are the high risk factors of GDM Among the mentioned high risk factors obesity is the one that can be controlled by taking low caloric diets and regular exercise. Thus both maternal and neonatal complications of GDM can be reduced by obstetricians with standard treatment consisting of individual dietary and lifestyle advice during pregnancy.

## Notes

***Source of Funding:*** Higher Education Commission.
